# Integrating Machine Learning with Human Knowledge

**DOI:** 10.1016/j.isci.2020.101656

**Published:** 2020-10-09

**Authors:** Changyu Deng, Xunbi Ji, Colton Rainey, Jianyu Zhang, Wei Lu

**Affiliations:** 1 Department of Mechanical Engineering, University of Michigan, Ann Arbor, MI 48109, USA; 2 Department of Materials Science & Engineering, University of Michigan, Ann Arbor, MI 48109, USA

**Keywords:** Computer Science, Artificial Intelligence, Human-Centered Computing

## Abstract

Machine learning has been heavily researched and widely used in many disciplines. However, achieving high accuracy requires a large amount of data that is sometimes difficult, expensive, or impractical to obtain. Integrating human knowledge into machine learning can significantly reduce data requirement, increase reliability and robustness of machine learning, and build explainable machine learning systems. This allows leveraging the vast amount of human knowledge and capability of machine learning to achieve functions and performance not available before and will facilitate the interaction between human beings and machine learning systems, making machine learning decisions understandable to humans. This paper gives an overview of the knowledge and its representations that can be integrated into machine learning and the methodology. We cover the fundamentals, current status, and recent progress of the methods, with a focus on popular and new topics. The perspectives on future directions are also discussed.

## Highlights

• Integrating knowledge into machine learning delivers superior performance
• Knowledge is categorized and its representations are presented
• Various methods to bridge human knowledge and machine learning are shown
• Suggestions on approaches and perspectives on future research directions are provided

## Graphical Abstract

**Figure:**
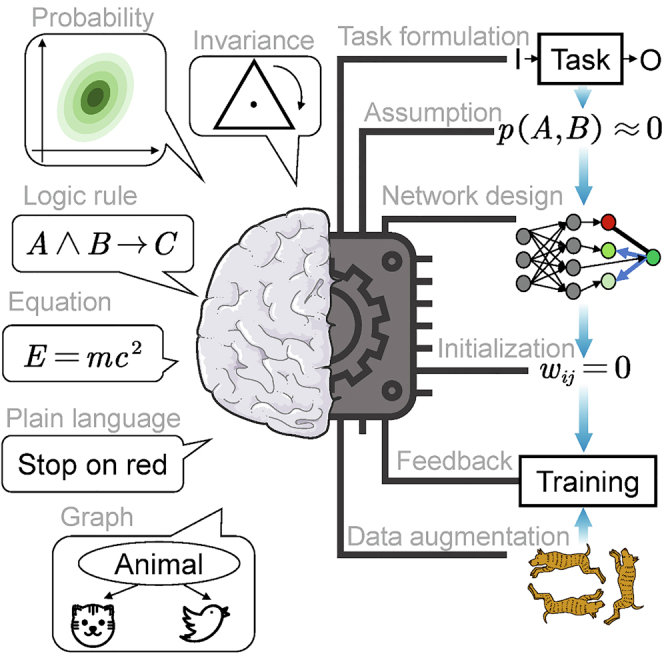

## Introduction

Machine learning has been heavily researched and widely used in many areas from object detection (Zou et al., 2019) and speech recognition (Graves et al., 2013) to protein structure prediction (Senior et al., 2020) and engineering design optimization (Deng et al., 2020; Gao and Lu, 2020; Wu et al., 2018). The success is grounded in its powerful capability to learn from a tremendous amount of data. However, it is still far from achieving intelligence comparable to humans. As of today, there have been few reports on artificial intelligence defeating humans in sensory tasks such as image recognition, object detection, or language translation. Some skills are not acquired by machines at all, such as creativity, imagination, and critical thinking. Even in the area of games where humans may be beaten, machine behaves more like a diligent learner than a smart one, considering the amount of data requirement and energy consumption. What is worse, pure data-driven models can lead to unintended behaviors such as gradient vanishing (Hu et al., 2018; Su, 2018) or classification on the wrong labels with high confidence (Goodfellow et al., 2014). Integrating human knowledge into machine learning can significantly reduce the data required, increase the reliability and robustness of machine learning, and build explainable machine learning systems.

Knowledge in machine learning can be viewed from two perspectives. One is “general knowledge” related to machine learning but independent of the task and data domain. This involves computer science, statistics, neural science, etc., which lays down the foundation of machine learning. An example is the knowledge in neural science that can be translated to improving neural network design. The other is “domain knowledge” which broadly refers to knowledge in any field such as physics, chemistry, engineering, and linguistics with domain-specific applications. Machine learning algorithms can integrate domain knowledge in the form of equations, logic rules, and prior distribution into its process to perform better than purely data-driven machine learning.

General knowledge marks the evolution of machine learning in history. In 1943, the first neuron network mathematical model was built based on the understanding of human brain cells (McCulloch and Pitts, 1943). In 1957, perceptron was invented to mimic the “perceptual processes of a biological brain” (Rosenblatt, 1957). Although it was a machine instead of an algorithm as we use today, the invention set the foundation of deep neuron networks (Fogg, 2017). In 1960, the gradients in control theory were derived to optimize the flight path (Kelley, 1960). This formed the foundation of backpropagation of artificial neural networks. In 1989, Q-learning was developed based on the Markov process to greatly improve the practicality and feasibility of reinforcement learning (Watkins and Dayan, 1992). In 1997, the concept of long short-term memory was applied to a recurrent neural network (RNN) (Hochreiter and Schmidhuber, 1997). The development of these algorithms, together with an increasing amount of available data and computational power, brings the era of artificial intelligence today.

Domain knowledge plays a significant role in enhancing the learning performance. For instance, experts' rating can be used as an input of the data mining tool to reduce the misclassification cost on evaluating lending applications (Sinha and Zhao, 2008). An accurate estimation of the test data distribution by leveraging the domain knowledge can help design better training data sets. Human involvement is essential in several machine learning problems, such as the evaluation of machine-generated videos (Li et al., 2018). Even in areas where machine learning outperforms humans, such as the game of Go (Silver et al., 2017), learning from records of human experience is much faster than self-play at the initial stage.

Knowledge is more or less reflected in all data-based models from data collection to algorithm implementation. Here, we focus on typical areas where human knowledge is integrated to deliver superior performance. In Section Knowledge and Its Representations, we discuss the type of knowledge that has been incorporated in machine learning and its representations. Examples to embed such knowledge will be provided. In Section Methods to Integrate Human Knowledge, we introduce the methodology to incorporate knowledge into machine learning. For a broad readership, we start from the fundamentals and then cover the current status, remarks, and future directions with particular attention to new and popular topics. We do not include the opposite direction, i.e. improving knowledge-based models by data-driven approaches. Different from a review on related topics (Rueden et al., 2019), we highlight the methods to bridge machine learning and human knowledge, rather than focusing on the topic of knowledge itself.

## Knowledge and Its Representations

Knowledge is categorized into general knowledge and domain knowledge as we mentioned earlier. General knowledge regarding human brains, learning process, and how it can be incorporated is discussed in Section Human Brain and Learning. Domain knowledge is specifically discovered, possessed and summarized by experts in certain fields. In some subject areas, domain knowledge is abstract or empirical, which makes it challenging to be integrated into a machine learning framework. We discuss some recent progresses on this form of knowledge in Section Qualitative Domain Knowledge. Meanwhile, the knowledge base is becoming more systematic and quantitative in various fields, particularly in science and engineering. We discuss how quantitative domain knowledge can be utilized in Section Quantitative Domain Knowledge.

### Human Brain and Learning

Machine learning uses computers to automatically process data to look for patterns. It mimics the learning process of biological intelligence, especially humans. Many breakthroughs in machine learning are inspired by the understanding of learning from fields such as neuroscience, biology, and physiology. In this section, we review some recent works that bring machine learning closer to human learning.

For decades, constructing a machine learning system required careful design of raw data transformation to extract their features for the learning subsystem, often a classifier, to detect or classify patterns in the input. Deep learning (LeCun et al., 2015) relaxes such requirements by stacking multiple layers of artificial neural modules, most of which are subject to learning. Different layers could extract different levels of features automatically during training. Deep learning achieved record-breaking results in many areas. Despite dramatic increase in the size of networks, the architecture of current deep neural networks (DNNs) with $10^{7}$ learnable weights is still much simpler than the complex brain network with $10^{11}$ neurons (Herculano-Houzel, 2009) and $10^{15}$ synapses (Drachman, 2005).

Residual neural networks (ResNets) (He et al., 2016), built on convolutional layers with shortcuts between different layers, were proposed for image classification. The input of downstream layers also contains the information from upstream layers far away from them, in addition to the output of adjacent layers. Such skip connections have been observed in brain cells (Thomson, 2010). This technique helps ResNets to win first place on the ImageNet Large Scale Visual Recognition Challenge (ILSVRC) 2015 classification task (He et al., 2016) and is widely used in other DNNs (Wu et al., 2019).

Dropout, motivated by “theory of the role of sex in evolution”, is a simple and powerful way to prevent overfitting of neural networks (Hinton et al., 2012; Srivastava et al., 2014). It is also analogous to the fact that neurons and connections in human brains keep growing and dying (breaking). At the training time, the connections of artificial neurons break randomly, and the remaining ones are scaled accordingly. This process can be regarded as training different models with shared weights. At the test time, the outputs are calculated by a single network without dropout, which can be treated as an average of different models. In recent years, dropout is proposed to be used at the test time to evaluate uncertainty (Gal and Ghahramani, 2016; Gal et al., 2017).

The focus of machine learning nowadays is on software or algorithms, yet some researchers start to redesign the hardware. Current computers, built on the von Neumann architecture, have a power consumption that is several orders of magnitude higher than biological brains. For example, International Business Machines Corporation (IBM)'s Blue Gene/P supercomputer simulated 1% of the human cerebral cortex and could consume up to 2.9 MW of power (Hennecke et al., 2012) while a human brain consumes only around 20 W (Modha, 2017). Neuromorphic systems were proposed and designed to improve energy efficiency. To name a few practical implementations, TrueNorth from IBM (Merolla et al., 2014; Modha, 2017) can solve problems from vision, audition, and multi-sensory fusion; Loihi from Intel (Davies et al., 2018) can solve the least absolute shrinkage and selection operator (LASSO) optimization orders of magnitude superior than the conventional central processing unit (CPU); NeuroGrid from Stanford University (Benjamin et al., 2014) offers affordable biological real-time simulations. In these brain-inspired systems, information is stored and processed at each neuron's synapses, which themselves are responsible for learning (Bartolozzi and Indiveri, 2007). There are various models to mimic neurons by circuits, such as the integrate-and-fire (I&F) model (Bartolozzi and Indiveri, 2007), the Hodgkin-Huxley model (Hodgkin and Huxley, 1952), and the Boolean logic gate design (Deshmukh et al., 2005). The majority of implementations use the I&F model, though their specific circuitry and behaviors vary (Nawrocki et al., 2016). As for materials, most systems utilize the standard silicon fabrication, which allows for integration with relatively mature technology and facilities. Recently, a few neuromorphic components or systems are made of new materials, such as organic or nanoparticle composites (Sun et al., 2018; Tuchman et al., 2020).

### Qualitative Domain Knowledge

Along with the neuroscience-inspired fast development of deep learning architecture, knowledge from specific domains enlightens innovative machine learning algorithms. In its primitive format, knowledge is descriptive, intuitive, and often expressed by plain language. Such knowledge is usually based on observation, inference, and induction. Here, we group it as qualitative domain knowledge which requires intensive engagement and interpretation by humans with sophisticated skills. Although there is no universal way to bridge machine learning and qualitative knowledge, qualitative knowledge adds unique insights into the machine learning framework by using customized strategies. This integration offers two primary advantages. For one thing, qualitative knowledge is explained mainly by experts, which means that it highly relies on subjective interpretation. Machine learning consolidates the stringency of expert knowledge so that it can be directly validated by the large amount of raw data. Therefore, the qualitative knowledge can be built on a more statistically rigorous base. For another thing, machine learning models, such as the DNN, are subject to interpretability issues. Using qualitative domain knowledge in machine learning helps dig into the underlying theoretical mechanisms.

Qualitative knowledge can be further divided into three subgroups according to the degree of quantification. Here, we name them as *Knowledge in Plain Language*, *Loosely Formed Knowledge*, and *Concretely Formatted Knowledge*.

#### Knowledge in Plain Language

Tremendous qualitative knowledge is well established in different disciplines, especially for social science. Sociology has been developed for thousands of years, with modern theory focusing on globalization and micro-marco structures (Ritzer and Stepnisky, 2017). Political scientists proposed institutional theories to profile current governments (Peters, 2019). These theories are usually in the form of plain language. Traditionally, machine learning is far from these domains. However, many empirical theories actually provide good intuition to understand and design better machine learning models. For example, machine learning researchers found that some widely used DNN models have “shape bias” in word recognition (Ritter et al., 2017). This means that the shape of characters is more important than color or texture in visual recognition. At the same time, research in development psychology shows that humans tend to learn new words based on similar shape, rather than color, texture, or size (Nakamura et al., 2012a). This coincidence provides a new theoretical foundation to understand how the DNN identifies objects. Conversely, some unfavorable biases, such as those toward race and gender, should be eliminated (DeBrusk, 2018).

#### Loosely Formed Knowledge

Qualitative knowledge can be pre-processed in ways that it is expressed in more numerical formats for use in machine learning. One example is that empirical human knowledge in social science can be inserted into machine learning through qualitative coding (Chen et al., 2018). This technique assigns inferential labels to chunks of data, enabling later model development. For example, social scientists in natural language processing (NLP) use their domain knowledge to group and structure the code system in the postprocessing step (Crowston et al., 2012). Moreover, qualitative coding is able to infer relations among latent variables. Human-understandable input and output are crucial for interpretable machine learning. If the input space contains theoretically justified components by humans, the mapping to output has logical meanings, which is much more preferred compared with pure statistical relationship (Liem et al., 2018).

In addition to social science, qualitative knowledge in natural science can be integrated into machine learning as well. For example, physical theories could guide humans to create knowledge-induced features for Higgs boson discovery (Adam-Bourdarios et al., 2015). Another strategy is to transfer language-based qualitative knowledge into numerical information. In computational molecular design (Ikebata et al., 2017), a molecule is encoded into a string including information such as element types, bond types, and branching components. The string is further processed by NLP models. The final strategy is to use experts to guide the learning process to identify a potential search direction. For instance, in cellular image annotation (Kromp et al., 2016), expert knowledge progressively improves the model through an online multi-level learning.

#### Concretely Formatted Knowledge

Although qualitative knowledge is relatively loose in both social and natural science, there are some formalized ways to represent it. For example, logic rules are often used to show simple relationships, such as implication (*A* → *B*), equivalence (*A* ↔ *B*), conjunction (*A* ∧ *B*), disjunction (*A* ∨ *B*), and so on. The simple binary relationship can be extended to include more entities by parenthesis association. It can be defined as a first order logic that each statement can be decomposed into a subject, a predicate, and their relationship. Logic rule–regularized machine learning models attract attention recently. For example, a “but” keyword in a sentence usually indicates that the clause after it dominates the overall sentiment. This first-order logic can be distilled into a neural network (Hu et al., 2016). In a material discovery framework called CombiFD, complex combinatorial *prioris* expressing whether the data points belong to the same cluster (Must-Link) or not (Cannot-Link) can be used as constraints in model training (Ermon et al., 2014).

Besides logic rules, invariance is another major format of qualitative knowledge which is not subject to change after transformation. An ideal strategy is to incorporate invariance in machine learning models, i.e., to build models invariant to input transformation, yet this is sometimes very difficult. Some details are shown in Section Symmetry of Convolutional Neural Networks. Besides models, one can leverage the invariant properties by preprocessing the input data. One way is to find a feature extraction method whose output is constant when the input is transformed within the symmetry space. For example, the Navier-Stokes equations obey the Galilean invariance. Consequently, seven tensors can be constructed from the velocity gradient components to form an invariant basis (Ling et al., 2016). The other way is to augment the input data based on invariance and feed the data to models (see Section Data Augmentation).

### Quantitative Domain Knowledge

In scientific domains, a large amount of knowledge has been mathematically defined and expressed, which facilitates a quantitative analysis using machine learning. In the following sections, three groups of quantitative knowledge, in terms of their representation formats, are discussed: equation-based, probability-based, and graph-based knowledge.

#### Equation-Based Knowledge

Equality and inequality relationships can be established by algebraic and differential equations and inequations, respectively. They are the predominant knowledge format in physics, mathematics, etc. At the same time, there is increasing amount of equation-based knowledge in chemistry, biology, engineering, and other experiment-driven disciplines. A great benefit of equations in aforementioned areas is that most variables have physical meanings understandable by humans. Even better, many of them can be measured or validated experimentally. The insertion and refinement of expert knowledge in terms of equations can be static or dynamic. Static equations often express a belief or truth that is not subject to change so that they do not capture the change of circumstance. Dynamically evolving equations, such as those used in the control area, are being used to express continuously updating processes. Equations in different categories play diverse roles in the machine learning pipeline, so equation-based knowledge can be further divided into subgroups according to their complexity.

The simplest format is a ground-truth equation, expressing consensus such as Mass=Density×Volume. Since it cannot be violated, this type of equation is usually treated as constraints to regularize the training process in the format of *Loss(x)* = *original_Loss(x)*+$\underset{i}{\sum }\lambda_{i}h_{i}\left(x\right)$, where $h_{i}\left(x\right)$ is the equation-enforced regularization term and $\lambda_{i}$ is the weight. For example, the object trajectory under gravity can be predicted by a convolutional neural network (CNN) without any labeled data, by just using a kinetic equation as the regularization term (Stewart and Ermon, 2017). The kinetic equation is easily expressed as a quadratic univariate equation of time or $h\left(t\right)=at^{2}+v_{0}t+h_{0}$. In another study, a robotic agent is designed to reach an unknown target (Ramamurthy et al., 2019). The solid body property enforces a linear relationship of segments, which serves as a regularizer in the policy architectures. The confidence of ground truth influences the degree of regularization through a soft or hard hyperparameter. For a complicated task, an expert must choose the confidence level properly.

At the second level, the equation has concrete format and constant coefficients, with single or multiple unknown variables and their derivatives. The relationship among those variables is deterministic. This means that the coefficients of these equations are state independent. Particularly, ordinary differential equation (ODE) and partial differential equation (PDE) belong to this category, which are being researched extensively within machine learning. They have the generalized form of $f\left(x_{1},...,x_{n},\frac{\partial u}{\partial x_{1}},...\right)$ = 0. Although only few differential equations have explicit solutions, as long as their formats can be determined by domain knowledge, machine learning can numerically solve them. This strategy inspires data-driven PDE/ODE solvers (Samaniego et al., 2020). Prior knowledge, such as periodicity, monotonicity, or smoothness of underlying physical process, is reflected in the kernel function and its hyperparameters.

In its most complicated format, equations may not fully generalize domain knowledge when the system has characteristics of high uncertainty, continuous updating, or ambiguity. The coefficients are state dependent or unknown functionals. These issues can be partially addressed by building a hybrid architecture of machine learning and PDE, in which machine learning helps predict or resolve such unknowns. For example, PDE control can be formulated as a reinforcement learning problem (Farahmand et al., 2017). The most extreme condition is that the form of coefficient/equation is unknown, but it can still be learned by machine learning purely from harnessing the experimental and simulation data. For example, governing equations expressed by parametric linear operators, such as heat conduction, can be discovered by machine learning (Raissi et al., 2017). Maximum likelihood estimation (MLE) with Gaussian process priors is used to infer the unknown coefficients from the noisy data. In another example, researchers propose to estimate nonlinear parameters in PDEs using quantum-behaved particle swarm optimization with Gaussian mutation (Tian et al., 2015). Inverse modeling can also be used to reconstruct functional terms (Parish and Duraisamy, 2016).

#### Probability-Based Knowledge

Knowledge in the form of probabilistic relations is another major type of quantitative knowledge. A powerful tool used in machine learning is Bayes' theorem which regulates the conditional dependence. We have prior knowledge of the relations of variables and their distributions. Given data, we could adjust some probabilities to fit observations.

Some machine learning algorithms have the intrinsic structure to capture probabilistic relations. For example, parameters of the conditional distribution in Bayesian network can be learned from data or directly from encoded knowledge (Flores et al., 2011; Masegosa and Moral, 2013). Domain knowledge can also be directly used to determine probabilities. For instance, gene relations help build optimal Bayesian classification by mapping into a set of constraints (Boluki et al., 2017). For instance, if gene g_2_ and g_3_ regulate g_1_ with *X*_*1*_ *=* 1 when *X*_*2*_ *=* 1 and *X*_*3*_ *=* 0 according to the domain knowledge, the constraint can be enforced by $P\left(X_{1}=1|X_{2}=1,\;X_{3}=0\right)=1.\;$

#### Graph-Based Knowledge

In both natural science and social science fields, a lot of knowledge has the “subject verb object” structure. A knowledge graph consists of entities and links among them. It can be expressed as a set of triples. The degree of their correlation can be numerically expressed and graphically visualized. Knowledge graphs are initially built by expert judgment from data. With growing size of data available, machine learning algorithms play an important role in constructing large knowledge graphs.

Google knowledge graph is an example that we access daily. It covers 570 million entities and 18 billion facts initially and keeps growing to have over 500 billion facts on ∼5 billion entities (Paulheim, 2017). For example, when you search basketball in Google, highly related NBA teams and stars will appear on the right. Another famous general knowledge-formed knowledge graph is ConceptNet, which connects words and phrases of natural language with labeled edges. It can be combined with word embeddings with a better understanding of the underlying meanings (Speer et al., 2016). Specific knowledge graphs are popular in different domains. In NLP, WordNet is a semantic network to link synonyms for highlighting their meanings rather than spelling. It can enhance the performance of search applications (Fellbaum, 2012). Medical and biologic fields have, for instance, MeSH (Lowe and Barnett, 1994). It is hierarchically organized vocabulary used for indexing, cataloging, and searching of biomedical and health-related information, upon which some machine learning models are built (Abdelaziz et al., 2017; Gan et al., 2019).

Knowledge graphs and machine learning mutually benefit each other. A graph may be incomplete when there are missing entities, relations, or facts and thus needs machine learning approaches to supplement the information. For example, knowledge graph can be trained together with tasks of recommendation to connect items (Cao et al., 2019). Human-computer interaction and knowledge discovery approach (Holzinger et al., 2013) can be used to identify, extract, and formalize useful data patterns from raw medical data. Meanwhile, knowledge graph helps human to understand the related fields to promote machine learning. For instance, a better understanding of biology and neuroscience leads to advanced machine learning algorithms and neuromorphic systems (Section Human Brain and Learning). Besides, machine learning models, such as neural networks, can be built upon knowledge graph, as is further discussed in Section Design of Neuron Connections.

## Methods to Integrate Human Knowledge

A complete process to design and implement a machine learning model consists of multiple steps. The first step is to formulate the appropriate tasks based on the goal. One needs to determine what the machine should learn, i.e., the inputs and outputs. After simplifying the problem by some assumptions, a model is built with unknown parameters to explain or correlate the inputs and outputs. Then, the model is initialized and trained by the collected data. After this, the machine learning model is ready for inference and evaluation. In practice, the process is not necessarily in such a chronological order but usually follows an iterative process. Some algorithms incorporate humans in the loop for feedback and evaluation. Human knowledge can be incorporated almost anywhere in this process. In the following sections, we review the methods to integrate human knowledge into machine learning. We organize them based on the sub-domains of machine learning field and group them according to their major contribution to the steps aforementioned. We should note that (1) there are numerous approaches, thus we focus on popular or emerging methods that could work efficiently across disciplines; (2) the methods are interwoven, namely, they may be used in several sub-domains and contribute to multiple steps; thus, we consider their main categories and detail them only in one place.

### Task Formulation

Machine learning models, such as neural networks and support vector machines, take an array of numbers in the form of vectors or matrices as inputs and make predictions. For a given goal, there remains flexibility for humans to formulate the task, i.e., to determine the inputs and outputs of the machine learning models. Humans could combine similar tasks based on their background and shared information (Section Multitask Learning). Domain knowledge is necessary to understand and make use of the similarity of tasks. Also, we need to carefully decide the inputs of machine learning models (Section Features) to best represent the essence of the tasks. We can leverage expert knowledge in the domain or some statistical tools when engineering these features.

#### Multitask Learning

Humans do not learn individual tasks in a linear sequence, but they learn several tasks simultaneously. This efficient behavior is replicated in machine learning with multitask learning (MTL). MTL shares knowledge between tasks so they are all learned simultaneously with higher overall performance (Ruder, 2017a). By learning the tasks simultaneously, MTL helps to determine which features are significant and which are just noise in each task (Ruder, 2017a). Human knowledge is used in MTL to determine if a group of tasks would benefit from being learned together. For example, autonomous vehicles use object recognition to drive safely and arrive at the intended destination. They must recognize pedestrians, signs, road lines, other vehicles, etc. Machine learning could be trained to recognize each object individually with supervised learning, but human knowledge tells us that the objects share an environment. The additional context increases accuracy as MTL finds a solution that fits all tasks. MTL has also been used extensively for facial landmark detection (Ehrlich et al., 2016; Ranjan et al., 2017; Trottier et al., 2017; Zhang et al., 2014) and has even contributed to medical research through drug behavior prediction (Lee and Kim, 2019; Mayr et al., 2016; Yuan et al., 2016) and through drug discovery (Ramsundar et al., 2015). Object recognition is a common use of MTL due to its proven benefits when used alongside CNNs (Girshick, 2015; Li et al., 2016).

**Figure 1:**
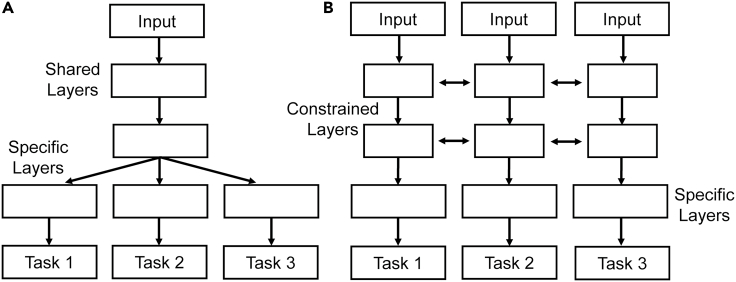
Illustration of Hard and Soft Parameter Sharing (A) hard parameter sharing. (B) soft parameter sharing. Redrawn from (Ruder, 2017a).

Currently, the two main methods of task sharing are hard and soft parameter sharing as shown in Figure 1. Hard parameter sharing is where some hidden layers are shared while the output layers remain task specific. In soft parameter sharing, each task has its own model and parameters, but the parameters are encouraged to be similar through the L2 norm regularization. Currently, hard parameter sharing is more common due to being more effective in reducing overfitting. Alternative methods to hard and soft parameter sharing have been proposed, such as deep relationship networks (Long et al., 2017), cross-stitch networks (Misra et al., 2016), and sluice networks (Ruder et al., 2019). Deep relationship networks use matrices to connect the task-specific layers so they also increase the performance alongside the shared layers. Cross-stitch networks attempt to find the optimal combination of shared and specific layers. Sluice networks combine several techniques to learn which layers should be shared. These methods aim to find a general approach that works broadly so that it can be easily used for all MTL problems. However, so far, the optimal task sharing method is different for each application. This means human knowledge on the application subject and on the various task sharing methods is a necessity to find the best method for the application. It has been found that MTL is unlikely to improve performance unless the tasks and the weighting strategies are carefully chosen (Gong et al., 2019). Continued research is needed for optimal strategies to choose and balance tasks.

Another important area in MTL is to gain benefits even in the case where only one task is important. Research shows that MTL can still be used in this situation by finding an appropriate auxiliary task to support the main task (Ruder, 2017a). Similar to finding the best task sharing method, significant human knowledge is needed to find an effective auxiliary task. Another approach is to use an adversarial auxiliary task which achieves the opposite purpose of the main task. By maximizing the loss function of the adversarial task, information can be gained for the main task. There are several other types of auxiliary tasks, but sometimes an auxiliary task is not even needed. In recent developments, MTL principles are utilized even in single task settings. Pseudo-task augmentation is where a single task is being solved; however, multiple decoders are used to solve the task in different ways (Meyerson and Miikkulainen, 2018). Each solving method is treated as a different task and implemented into MTL. This allows a task to be solved optimally as each method of solving the task learns from the other methods.

#### Features

“Features” in machine learning refer to variables that represent the property or characteristic of the observations. They could be statistics (e.g., mean, deviation), semantic attributes (e.g., color, shape), transformation of data (e.g, power, logarithm), or just part of raw data. “Feature engineering” is the process to determine and obtain input features to optimize machine learning performance. In this section, we will discuss the approaches in this process.

Feature engineering, especially feature creation, heavily relies on human knowledge and experience in the areas. For instance, in a credit card fraud detection task, there are many items associated with each transaction, such as transaction amount, merchant ID, and card type. A simple model treats each transaction independently and classifies the transactions by eliminating unimportant items (Brause et al., 1999). Later, people realize that costumer spending behaviors matter as well. Then, their indicators, such as transactions during the last give number of hours and countries, are aggregated (Whitrow et al., 2009). This methodology is further amended by capturing transaction time and its periodic property (Bahnsen et al., 2016). We could see from this example that it is an iterative process that interplays with feature engineering and model evaluation (Brownlee, 2014). Although the guidelines vary with specific areas, a rule of thumb is to find the best representation of the sample data to learn a solution.

In addition to domain knowledge, there are some statistical metrics in feature selection widely used in different areas. There are mainly two types of ideas (Ghojogh et al., 2019). One is called filter methods, which rank the features by their relevance (correlation of features with targets) or redundancy (whether the features share redundant information) and remove some by setting a threshold. This type includes linear correlation coefficient, mutual information, consistency-based filter (Dash and Liu, 2003), and many others. The other type is called wrapper methods. These methods train and test the model during searching. They look for the subset of features that correspond to the optimal model performance. Since the number of combinations grow exponentially with the number of features, it is a non-deterministic polynomial-time (NP) hard problem to find the optimal subset. One searching strategy, sequential selection methods, is to access the models sequentially (Aha and Bankert, 1996). The other strategy is to implement metaheuristic algorithms such as the binary dragonfly algorithm (Mafarja et al., 2017), genetic algorithm (Frohlich et al., 2003), and binary bat algorithm (Nakamura et al., 2012b). Wrapper methods can be applied to simple models instead of the original ones to reduce computation. For instance, Boruta (Kursa and Rudnicki, 2010) uses a wrapper method on random forests, which in essence can be regarded as a filter method. In addition to these two types, there exist other techniques such as embedded methods, clustering techniques, and semi-supervised learning (Chandrashekar and Sahin, 2014).

“Feature learning”, also called “representation learning”, is a set of techniques that allow machine to discover or extract features automatically. Since the raw data may contain redundant information, we normally want to extract features with lower dimension, i.e., to find a mapping $R^{d}\to R^{p}$ where p≤d and usually p≪d. Thus, these methods are sometimes referred to as dimension reduction. Traditional ways are statistical methods to extract features. Unsupervised (unlabeled data) methods include principal component analysis (Wold et al., 1987), maximum variance unfolding (Weinberger and Saul, 2006), Laplacian eigenmap (Belkin and Niyogi, 2003; Chen et al., 2010), and t-distributed stochastic neighbor embedding (t-SNE) (Maaten and Hinton, 2008). Supervised (labeled data) methods include Fisher linear discriminant analysis (Fisher, 1936), its variant Kernel Fisher linear discriminant analysis (Mika et al., 1999), partial least squares regression (Dhanjal et al., 2008), and many approaches on supervised principal component analysis (Bair and Tibshirani, 2004; Barshan et al., 2011; Daniušis et al., 2016). Recent works are mostly based on neural networks, such as autoencoders (Baldi, 2012), CNNs, deep Boltzman machines (Salakhutdinov and Hinton, 2009). Even though these methods extract features automatically, prior knowledge can still be incorporated. For instance, to capture the features of videos with slow moving objects, we can represent the objects by a group of numbers (such as their positions in space and the pose parameters) rather than by a single scalar, and these groups tend to move together (Bengio et al., 2013; Hinton et al., 2011). Or we can deal with the moving parts and the static background separately (Li et al., 2018). Another example, which applies the principal of Multitask Learning, is to train an image encoder and text encoder simultaneously and correlate their extracted features (Reed et al., 2016). Other strategies to integrate knowledge include data manipulation and neural network design, as will be discussed in the following sections.

### Model Assumptions

Machine learning models are built upon assumptions or hypotheses. Since “hypothesis” in machine learning field commonly refers to model candidates, we use the word “assumption” to denote explicit or implicit choices, presumed patterns, and other specifications on which models are based for simplification or reification. The assumptions could be probabilistic or deterministic. We first introduce probabilistic assumptions in Sections Preliminaries of Probabilistic Machine Learning, Variable Relation and Distribution, and then briefly discuss Deterministic Assumptions.

#### Preliminaries of Probabilistic Machine Learning

Mathematically, what are we looking for when we train a machine learning model? From the perspective of probability, two explanations have been proposed as shown in Equations (1) and (2) (Murphy, 2012). One is called MLE (maximum likelihood estimation) which means that the model maximizes the likelihood of the training data set,

(Equation 1) $$\theta_{MLE}=\underset{\theta }{\text{argmax}}\;p\left(D|\theta \right)$$

where D is the training data set and p(D|θ) is the probability of data provided by the machine learning model whose parameter is θ. The machine learning model estimates the probability of observation, while the training process tries to find the parameter which best accords with the observation. The specific form of D depends on models. For example, in supervised learning, we can rewrite the form as p(Y|X,θ), i.e., the probability of label Y given input X and model parameters θ, and we regard the label with the highest probability as the model's prediction.

The other strategy, maximum a posteriori (MAP), means to maximize the posterior probability of the model parameter,

(Equation 2) $$\theta_{MAP}=\underset{\theta }{\text{argmax}}\;p\left(\theta |D\right)=\underset{\theta }{\text{argmax}}\;\frac{p\left(D|\theta \right)p\left(\theta \right)}{p\left(D\right)}=\underset{\theta }{\text{argmax}}\;p\left(D|\theta \right)p\left(\theta \right)$$

MAP takes into account p(θ), the *prior* distribution of θ. From the equations above, we can observe that MLE is a special case of MAP where p(θ) is uniform.

#### Variable Relation

A variable could be an instance (data point), a feature, or a state of the system. An assumption on variable relation used in almost all models is the independence of data instances; therefore, the probability of data sets is equal to the product of instance probabilities. For instance, in unsupervised learning we have $p(X|\theta )=\underset{i}{\prod }p(x^{\left(i\right)}|\theta )$.

When the variables are related, we could simplify the likelihood function by assuming partial independence. For instance, in image generation models, pixels are correlated to each other; thus, an image x is associated with all pixels $x_{i}$ (i=1,2,…). Pixel RNN (Oord et al., 2016a) assumes that pixel $x_{i}$ is only correlated to its previous pixels and independent of those afterward:

(Equation 3) $$p\left(x\right)=p\left(x_{1},x_{2},...,x_{N}\right)\approx \overset{N}{\underset{i=1}{\prod }}p\left(x_{i}|x_{1},x_{2},...,x_{i-1}\right)$$

Under this assumption, pixels are generated sequentially by an RNN. As a simpler implementation, we can pay more attention to the local information and calculate a pixel value by the previous ones close to it (Oord et al., 2016b).

In the stochastic process of time series, Equation (3) is commonly used, and $x_{i}$ denotes the variable value at time i. In this context, the approximation is more of knowledge than an assumption since it is hard to imagine that the future would impact the past. It can be simplified further by assuming that future probabilities are determined by their most recent values $p\left(x_{i}\left|x_{1},x_{2},...,x_{i-1}\right)\approx p\left(x_{i}\right|x_{i-1}\right)$ and then the variables $x_{i}$, called *states*, form a Markov chain. A more flexible model, hidden Markov model (HMM), treats the states as hidden variables and the observations have a conditional probability distribution given the states. HMM is widely used in stock market, data analysis, speech recognition, and so forth (Mor et al., 2020). Such Markov property is also presumed in reinforcement learning (Section Reinforcement Learning), where the next state is determined by the current state and action.

In addition to sequential dependence, the relation between variables can be formulated as a directed acyclic graph or a Bayesian network in which nodes represent the variables and edges represent the probabilistic dependencies. Learning the optional structure of a Bayesian network is NP-hard problem, which means that it requires a huge amount of computation and may not converge to global optima. Prior knowledge of the variables can be incorporated by their dependencies, such as the existence or absence of an edge and even the probability distribution (Su et al., 2014; Xu et al., 2015). The results of learned networks get improved while computational cost is reduced.

#### Distribution

The distribution of variables is unknown and has to be assumed or approximated by sampling. A very popular and fundamental distribution is the Gaussian distribution, a.k.a. normal distribution. Its popularity is not groundless; instead, it is based on the fact that the mean of a large number of independent random variables, regardless of their own distributions, tends toward Gaussian distribution (central limit theorem). The real-world data are composed of many underlying factors, so the aggregate variables tend to have a Gaussian distribution in nature. Some models are named after it, such as Gaussian process (Ebden, 2015) and Gaussian mixture model (Shental et al., 2004). Independent component analysis (ICA) decomposes the observed data into several underlying components and sources (Hyvärinen, 2013). ICA assumes that the original components are non-Gaussian. In linear regression models, the least square method can be derived from Equation (1) while assuming the output Y has a Gaussian distribution with a mean of $\theta^{T}X$, namely $p\left(D\left|\theta \right)=p\left(Y\right|X,\theta \right)=N\left(\theta^{T}X,\sigma^{2}\right)$ where σ is the standard deviation. Further, by assuming the prior p(θ) to be Gaussian with zero mean, regularized linear regression can be derived from Equation (2), whose loss function is penalized by the sum of the squares of the terms in θ.

Besides Gaussian, other types of distribution assumptions are applied as well. Student-t process is used in regression to model the priors of functions (Shah et al., 2014). In many Bayesian models, priors must be given through either the training data or manual adjustment. Inaccurate priors would cause systemic errors of the models. For instance, in spam email classification by naive Bayesian classifiers, if assuming uniform distribution of spam and non-spam (50/50), then the classifier is prone to report a non-spam email as spam when applying it to real life where the ratio of spam emails is much smaller, say 2%. Conversely, assuming a 2% spam ratio, the classifier would miss spam emails when applying it to email accounts full of spam emails. Although methods have been proposed to alleviate this issue (Frank and Bouckaert, 2006; Rennie et al., 2003), classifiers would benefit from appropriate priors. Such training-testing mismatch problem also occurs in other machine learning models. To the best of our knowledge, there is no perfect solution to this problem; thus, the most efficient way is to design a distribution of training data close to test scenarios which requires knowledge from experts.

#### Deterministic Assumptions

Deterministic assumptions describe the properties and relations of objects. Some deterministic assumptions can also be expressed in a probabilistic way, such as variable independence. In addition to these, many are encoded in the “hypothesis space”: for an unknown function f:X→Y, we use a machine learning model to approximate the target function. Hypothesis space is the set of all the possible functions. This is the set from which the algorithm determines the model which best describes the target function according to the data. For instance, in artificial neural networks, the configuration of the networks, e.g., the number of layers, activation functions, and hyperparameters, is determined priorly. Then, all combinations of weights span the hypothesis space. Through training, the optimal combination of weights is calculated. The design of networks can be integrated with human knowledge, which is elaborated in the section below.

### Network Architecture

Artificial neural network has been proved to be a very powerful tool to fit data. It is extensively used in many machine learning models especially after the emergence of deep learning (LeCun et al., 2015). Although ideally neural networks could adapt to all functions, adding components more specific to the domains can boost the performance. As mentioned in Section Deterministic Assumptions, the architecture of network regulates the hypothesis space. Using a specialized network reduces the size of hypothesis space and thus generalizes better with less parameters than universal networks. Therefore, we want to devise networks targeting at data and tasks.

There have been network structures proposed for specific tasks. For instance, RNNs are applied to temporal data, such as language, speech, and time series; capsule neural networks are proposed to capture the pose and spatial relation of objects in images (Hinton et al., 2011). In the following contents, we elaborate how symmetry is used in CNNs and how to embed different knowledge via customizing the neuron connections.

#### Symmetry of Convolutional Neural Networks

Symmetry, in a broad sense, denotes the property of an object which stays the same after a transformation. For instance, the label and features of a dog picture remain after rotation. In CNNs, symmetry is implemented by two concepts, “invariance” and “equivariance”. Invariance means the output stays the same when the input changes, i.e., f(Tx)=f(x) where x denotes the input, T denotes the transformation, and f denotes the feature mapping (e.g. convolution calculation). Equivariance means the output preserves the change of the input, i.e., $f\left(Tx\right)=T^{\prime }\left[f\left(x\right)\right]$ where $T^{\prime }$ is another transformation that could equal T. CNNs are powerful models for sensory data and especially images. A typical CNN for image classification is composed of mostly convolution layers possibly followed by pooling layers and fully connected ones for the last few layers. Convolution layers use filters to slide through images and calculate inner products with pixels to extract features to analyze each part of images. Such weight sharing characteristic not only greatly reduces the number of parameters but also provides them with inherent translation equivariance: the convolution output of a shifted image is the same as the shifted output of the original image. Basically, the CNN relies on convolution layers for equivariance and fully connected layers for invariance.

The inherent translation equivariance is limited: there are reports showing that the confidence of correct labels would decrease dramatically even with shift unnoticeable by human eyes. It is ascribed to the aliasing caused by down-sampling (stride) commonly used on convolution layers, and a simple fix is to add a blur filter before down-sampling layers (Zhang, 2019). Other symmetry groups are also considered, such as rotation, mirror, and scale. The principle of most works is to manipulate filters since they compute faster than data transformation. A simple idea is to use symmetric filters such as circular harmonics (Worrall et al., 2017) for rotation or log-radial harmonics (Ghosh and Gupta, 2019) for scale. Such equivariance or invariance is local, that is, only each pixel-filter operation has the symmetry property while the whole layer output, composed of multiple operations, does not. A global way is to traverse the symmetry space and represent with exemplary points (control points) (Cohen and Welling, 2016). For instance, in the previous dog example, we could rotate the filter by 90, 180, and 270° to calculate the corresponding feature maps. Thus, we can approximate the equivariance of rotation. Kernel convolution can be used to control the extent of symmetry, e.g. to distinguish “6” and “9” (Gens and Domingos, 2014). Overall, although invariant layers (Kanazawa et al., 2014) can also be constructed to incorporate symmetry, equivariant feature extraction as intermediate layers is preferred in order to preserve the relative pose of local features for further layers.

#### Design of Neuron Connections

By utilizing the knowledge of invariance and equivariance in the transformation, the CNN preforms well in image classification especially with some designed filters. From another perspective, we can also say that the CNN includes the knowledge of graphs. Imagine a pixel in a graph, which is connected to the other pixels around it. By pooling and weights sharing, the CNN generalizes the information of the pixel and its neighbors. This kind of knowledge is also applied in a graph neural network (GNN) (Gori et al., 2005; Wu et al., 2020) where the node's state is updated based on embedded neighborhood information. In the GNN, the neighbors are not necessarily the surrounding pixels but can be defined by designers. Thus, the GNN is able to represent the network nodes as low-dimensional vectors and preserve both the network topology structure and the node content information. Furthermore, the GNN can learn more informative relationship through differential pooling (Ying et al., 2018) and the variational evaluation-maximization algorithm (Qu et al., 2019).

Not only is the knowledge of general connections between nodes used in network design but also the specific relational knowledge in connections is beneficial. Encoding the logic graph (Fu, 1993) and hierarchy relationships (Deng et al., 2014) into the architecture, such as “A∧B→C” and “a ∈ A”, is also one way to build neural networks with Graph-Based Knowledge. With these encoding methods, some of the rules are learned from data and part of them are enforced by human knowledge. This idea is reflected in the cooperation of symbolic reasoning and deep learning, which is getting increasingly popular recently (Garnelo and Shanahan, 2019; Mao et al., 2019; Segler et al., 2018). Symbolic reasoning or symbolic artificial intelligence (AI) (Flasiński, 2016; Hoehndorf and Queralt-Rosinach, 2017) is a good example of purely utilizing graph-based knowledge where all the rules are applied by humans and the freedom of learning is limited, while in deep learning, the rules are automatically learned from data. Building symbolic concept into neural networks helps increase the network's interpreting ability and endow the networks with more possibilities, allowing more interactions between labels, for instance.

Aside from the knowledge of graphs, Equation-Based Knowledge can also be united with deep learning. For example, a general data-driven model can be added to a first-principle model (Zhou et al., 2017). The add-on neural networks will learn the complex dynamics which might be impossible to identify with pure physical models, e.g. learning the close-to-ground aerodynamics in the drone landing case (Shi et al., 2019). Another example in utilizing equation-based knowledge is using optimization equations in the layers through OptNet (Amos and Kolter, 2017), a network architecture which allows learning the necessary hard constraints. In these layers, the outputs are not simply linear combinations plus nonlinear activation functions but solutions (obtained by applying Karush-Kuhn-Tucker conditions) to constrained optimization problems based on previous layers.

In speech recognition, the words in a sentence are related, and the beginning of the sentence may have a huge impact on interpretation. This induces delays and requires accumulating information over time. Also, in many dynamical systems, especially those involving human reaction time, the effects of delays are important. The knowledge of delay is then introduced in the design of neural networks. For example, time-delay neural networks (Waibel et al., 1989) take information from a fixed number of delayed inputs and thus can represent the relations between sequential events. Neural networks with trainable delay (Ji et al., 2020) utilize the knowledge of delay's existence and learn the delay values from data. RNN (Graves et al., 2013; Zhang et al., 2019) is a network architecture in which neurons feedback in a similar manner to dynamical systems, where the subsequent state depends on the previous state. Through inferring the latent representations of states instead of giving label to each state, the RNN can be trained directly on text transcripts of dialogs. However, these end-to-end methods often lack constraints and domain knowledge which may lead to meaningless answers or unsafe actions. For instance, if a banking dialog system does not require the username and password before providing account information, personal accounts could be accessed by anyone. A hybrid code network (HCN) (Williams et al., 2017) is proposed to address this concern. The HCN includes four components: entity extraction module, RNN, domain-specific software, and action templates. The RNN and domain-specific software maintain the states, and the action templates can be a textual communication or an API call. This general network allows experts to express specific knowledge, achieves the same performance with less data, and retains the benefits of end-to-end training.

### Data Augmentation

Machine learning, especially deep learning, is hungry for data. The problems with insufficient data include overfitting where the machine generalizes poorly and class imbalance where the machine does not learn what we want because real-world data sets only contain a small percentage of “useful” examples (Salamon and Bello, 2017). These problems can be addressed by data augmentation, a class of techniques to artificially increase the amount of data with almost no cost. The basic approaches are transforming, synthesizing, and generating data. From the perspective of knowledge, it teaches machine invariance or incorporates knowledge-based models. Some papers do not consider simulation as data augmentation, but we will discuss it here since in essence these techniques are all leveraging human knowledge to generate data. Data augmentation needs more computation than explicit ways (e.g. Section Symmetry of Convolutional Neural Networks), but it is widely used in many areas such as Image, Audio, time series (Wen et al., 2020), and NLP (Fadaee et al., 2017; Ma, 2019) owing to its flexibility, simplicity, and effectiveness. It is even mandatory in unsupervised representation learning (Chen et al., 2020; He et al., 2020).

#### Image

**Figure 2:**
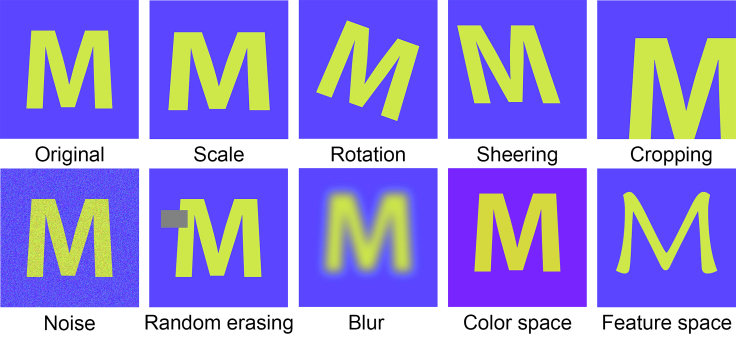
Illustration of Image Augmentation Techniques

Some representative data augmentation techniques for images are illustrated in Figure 2. A fundamental approach is to apply affine transformation to geometries, i.e., cropping, rotating, scaling, shearing, and translating. Noise and blur filters can be injected for better robustness. Elastic distortions (Simard et al., 2003), designed for hand-written character recognition, are generated using a random displacement field in image space to mimic uncontrolled oscillations of muscles (Wong et al., 2016). Random erasing (Zhong et al., 2020) is analogous to dropout except that it is applied to input data instead of network architecture. Kernel filters are used to generate blurred or sharpened images. Some kernels (Kang et al., 2017) can swap the rows and columns in the windows. This idea of “swapping” is similar to another approach, mixing images. There are two ways to mix images, one is cropping and merging different parts of images (Summers and Dinneen, 2019), the other is overlapping images and averaging their pixel values (Inoue, 2018). They have both been demonstrated to improve performance, though the latter is counterintuitive.

Another perspective is to change the input data in color space (Shorten and Khoshgoftaar, 2019). Images are typically encoded by RGB (red, green, blue) color space, i.e. represented by three channels (matrices) indicating red, green, and blue. These values are dependent upon brightness and lightning conditions. Therefore, color space transformation, also called photometric transformation, can be applied. A quick transformation is to increase or decrease the pixel values of one or three channels by a constant. Other methods are, for instance, setting thresholds of color values and applying filters to change the color space characteristics. Besides RGB, there are other color spaces such as CMY (cyan, magenta, yellow), HSV (hue, saturation, value which denotes intensity), and CIELab. The performance varies with color spaces. For example, a study tested four color spaces in a segmentation task and found that CMY outperformed the others (Jurio et al., 2010). It is worthy to note that human judgment is important in the freedom of color transformation since some tasks are sensitive to colors, e.g. distinguishing between water and blood. In contrast to augmentation, another direction to tackle color variance is to standardize the color space such as adjusting the white balance (Afifi and Brown, 2019).

Deep learning can be used to generate data for augmentation. One way is adversarial training which consists of two or more networks with contrasting objectives. Adversarial attacking uses networks to learn augmentations to images that result in misclassifications of their rival classification networks (Goodfellow et al., 2014; Su et al., 2019). Another method is generative models, such as generative adversarial networks and variational auto-encoders. These models can generate images to increase the amount of data (Lin et al., 2018). Style Transfer (Gatys et al., 2015), best known for its artistic applications, serves as a great tool for data augmentation (Jackson et al., 2019). In addition to images in the input space, data augmentation can also be applied to the feature space, i.e., the intermediate layers of neural networks (Chawla et al., 2002; DeVries and Taylor, 2017).

#### Audio

There are many types of audio, such as music and speech, and accordingly many types of tasks. Although augmentation method varies with audio types and tasks, the principles are universal.

Tuning is frequently used by music lovers and professionals to play or post-process music. There is a lot of mature software for it. They can stretch time to shift pitch or change the speed without pitch shifting. More advanced tuning involves reverberation (sound reflection in a small space), echo, saturation (non-linear distortion caused by overloading), gain (signal amplitude), equalization (adjusting balance of different frequency components), compression, etc. These methods can all be used in audio data augmentation (Mignot and Peeters, 2019; Ramires and Serra, 2019). Unfavorable effects in tuning, such as noise injection and cropping, are used as well in audio data augmentation.

An interesting perspective is to convert audio to images on which augmentations are based. Audio waveforms are converted to spectrograms which represent the intensity of a given frequency (vertical axis) at a given time (horizontal axis). Then, we can modify the “image” by distorting in the horizontal direction, blocking several rows, or blocking several columns (Park, 2019). These augmentations help the networks to be robust against, respectively, time direction deformations, partial loss of frequency channels, or partial loss of temporal segments of the input audio. This idea was extended by introducing more policies such as vertical direction distortion (frequency warping), time length control, and loudness control (Hwang et al., 2020). Other techniques used in image augmentation, e.g., rotation and mixture, are attempted as well (Nanni et al., 2020).

#### Simulation

As humans create faster and more accurate knowledge-based models to simulate the world, using simulations to acquire a large amount of data becomes an increasingly efficient method for machine learning. The primary advantage of simulations is the ability to gather a large amount of data when doing so experimentally would be costly, time consuming, or even dangerous (Ruder, 2017b; Ruiz et al., 2018). In some cases, acquiring real-world data may even be impossible without already having some training through simulations.

One application that conveys the important role of human knowledge in simulation data is computer vision. Humans can use their visual knowledge to develop powerful visual simulations to train computers. Autonomous vehicles, for instance, can be trained by simulated scenarios. They can learn basic skills before running on the road and can also be exposed to dangerous scenes that are rare naturally. Open-source simulation environments and video games such as Grand Theft Auto V (Martinez et al., 2017) can be used to reduce the time and money required to build simulations. Besides autonomous vehicles, simulation data have been used for computer vision in unique works such as cardiac resynchronization therapy (Giffard-Roisin et al., 2018), injection molding (Tercan et al., 2018), and computerized tomography (CT) scans (Holmes et al., 2019). Each of these applications requires thorough human knowledge of the subject. Lastly, robotics is a field where simulation data are expected to play a significant role in future innovation. Training robotics in the real world is too expensive, and the equipment may be damaged (Shorten and Khoshgoftaar, 2019). By incorporating human models, simulation data can allow robotics to be trained safely and efficiently.

Improvement through future research will accelerate the adoption of this technique for more tasks. Human experience and knowledge-based models will make simulations in general more realistic and efficient. Therefore, simulation data will become even more advantageous for training. At the same time, data-driven research aims to find optimal simulations that provide the most beneficial data for training the real-world machine. For instance, reinforcement learning is used to quickly discover and converge to simulation parameters that provide the data which maximizes the accuracy of the real-world machine being trained (Ruiz et al., 2018). While data-driven methods will reduce the human knowledge necessary for controlling the simulation, they will not replace the necessity of human knowledge for developing the simulation in the first place.

### Feedback and Interaction

As humans, we gain knowledge mostly from interactions with the environment. In some algorithms, the machine is designed to interact with humans. Including human-in-loop (Holzinger, 2016; Holzinger et al., 2019) can help interpret the data, promote the efficiency of learning, and enhance the performance.

A typical method that demonstrates how machines can interact with the environment, including humans and preset rules, is discussed in Section Reinforcement Learning. Knowledge can also be injected through the rewards as well. In Section Active Learning, machines may ask humans for data labeling or distribution. In Section Interactive Visual Analytics, machines interact with humans by bringing new knowledge through visualization while seeking manual tuning.

#### Reinforcement Learning

Reinforcement learning is a goal-directed algorithm (Sutton and Barto, 2018) which learns the optimal solution to a problem by maximizing the benefit obtained in interactions with the environment.

In reinforcement learning, the component which makes decisions is called “agent” and everything outside and influenced by the agent is “environment”. At time t, the agent has some information about the environment which can be denoted as a “state” $S_{t}$. According to the information, it will take an *action* $A_{t}$ under a *policy* $\pi_{t}\left(a|s\right)$, which is the probability of choosing $A_{t}=a$ when $S_{t}=s$. This action will lead to the next state $S_{t+1}$ and a numerical *reward* $R_{t+1}$. The transition between two states is given by interactions with the environment and is described by the environment model. If the probability of transition between states is given, we can solve the problem by applying model-based methods. When the model is unknown, which is usually the case in real life, we can also learn the model from the interactions and use the learned model to simulate the environmental behaviors if interactions are expensive.

The agent learns the optimal policy by maximizing the accumulated reward $G_{t}$ (usually in episodic problems and is called return) or average reward (in continuing problems). In general, the collected rewards can be evaluated by state value $V_{\pi }\left(s\right):=E_{\pi }\left[G_{t}|S_{t}=s\right]$ or action value $Q_{\pi }\left(s,a\right):=E_{\pi }\left[G_{t}|S_{t}=s,A_{t}=a\right]$. Based on different policy evaluation approaches, the methods used in reinforcement learning can be categorized into two types. One type is to evaluate the policy by the value function $V_{\pi }\left(s\right)$ or $Q_{\pi }\left(s,a\right)$. The value function is estimated through table (tabular methods) or approximator (function approximation methods). In the tabular methods, the exact values of those states or state-action pairs are stored in a table; thus, the tabular methods are usually limited by computation and memory (Kok and Vlassis, 2004). Function approximation (Xu et al., 2014) provides a way to bypass the computation and memory limit in high-dimensional problems. The states and actions are generalized into different features, then the value functions become functions of those features, and the weights in the function are learned through interactions. The other type is to evaluate the policy directly by an approximator. Apart from learning the value functions, an alternative approach in reinforcement learning is to express the policy with its own approximation, which is independent of the value function. These kinds of methods are called policy gradient methods, including actor-critic methods which learn approximations to both policy and value functions (Silver et al., 2014; Sutton et al., 2000). With these methods, the agent learns the policy directly.

Feedback from the human or the environment as a reward in reinforcement learning is essential since it is a goal-directed learning algorithm. There are many tasks for which human experience remains useful, and for those tasks, it would be efficient and preferable to obtain the knowledge from humans directly and quickly. Humans can participate in the training process of reinforcement learning in two ways, one is to indirectly shape the policy by constructing the reward function, while the other is to directly intervene with the policy during learning. In the former way, when the goal of some tasks is based on human satisfaction, we need humans to give the reward signal and ensure that the agent fulfills the goal as we expect (Knox and Stone, 2008). Including human rewards, the policy is pushed indirectly toward the optimal one under the human's definition, and the learning process is sped up (Loftin et al., 2016). Aside from giving reward manually after each action, human knowledge can also be used to design the reward function, e.g. give more positive weights to those important indicators in multi-goal problems (Hu et al., 2019). A recent work summarizes how to inject human knowledge into a tabular method with reward shaping (Rosenfeld et al., 2018). In the other way, guidelines from humans directly exist in the policy. Human feedback can modify the exploration policy of the agent and participate in the action selection mechanism (Knox and Stone, 2010, 2012). The feedback on policy can be not only a numerical number but also a label on the optimal actions (Griffith et al., 2013). By adding the label, human feedback changes the policy directly instead of influencing the policy through rewards. Recent works show that human feedback also depends on the agent's current policy which enables useful training strategies (MacGlashan et al., 2017), and involving humans in the loop of reinforcement learning gives improvement in learning performance (Lin et al., 2017).

#### Active Learning

**Figure 3:**
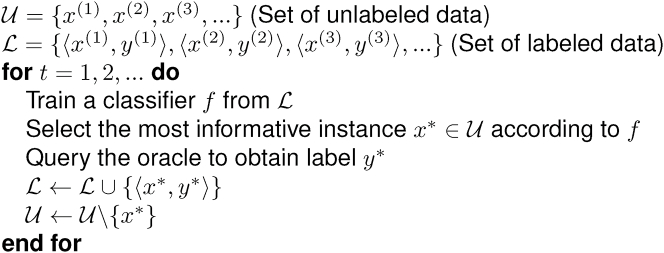
Pseudocode of an Active Learning Example Rephrased from (Settles, 2012)

Active learning, by selecting partial data to be annotated, aims to resolve the challenge that labeled data are more difficult to obtain than unlabeled data. During the training process, the learner poses “queries” such as some unlabeled instances to be labeled by an “oracle” such as a human. An example of active learning algorithm for classification is shown in Figure 3. Initially, we have some labeled data and unlabeled data. After training a model based on the labeled data, we can search for the most informative unlabeled data and query the oracle to obtain its label. Eventually, we can have an excellent classifier with only few additional labeled data. In this scenario, the learner makes a decision on the query after evaluating all the instances; thus, it is called “pool-based sampling”. Other scenarios include “stream-based selective sampling” (each unlabeled data point is examined one at a time with the learner evaluating the informativeness and deciding whether to query or discard) and “query synthesis” (the learner synthesizes or creates its own instance).

**Figure 4:**
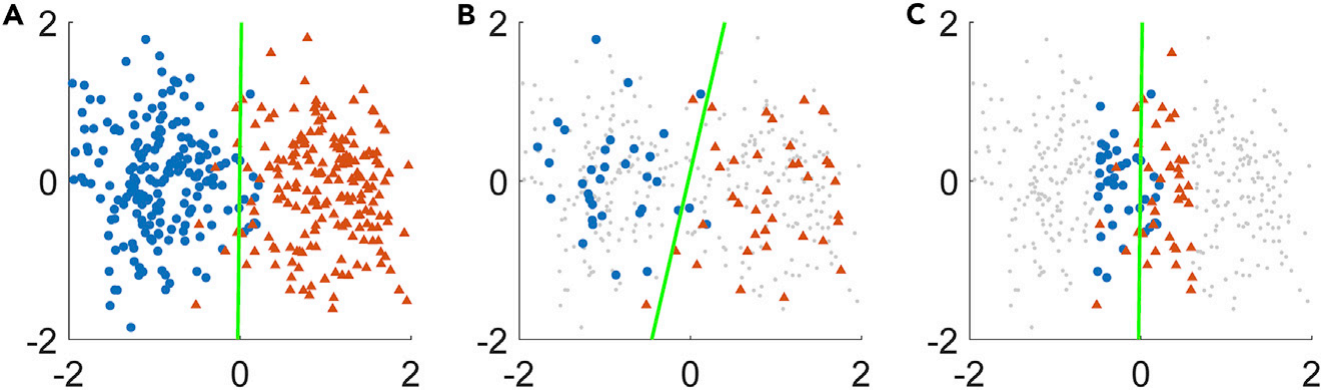
An Illustration of Active Learning: Choosing Data to Inquire for Better Estimation When Labeled Data Are Not Sufficient Data shown are randomly generated from two Gaussian distributions with different means. Drawn based on the concept in (Settles, 2012). (A) Correct labels of the binary classification problem. The line denotes the decision boundary. (B) A model trained by random queries. (C) A model trained by active queries.

There are many ways to define how informative a data point is, namely, “query strategies” vary. As illustrated in Figure 4, effective query strategies help the trained model outperform the one trained by random sampling. Therefore, it is a major issue in active learning to apply optimal query strategy, which has the following categories (Settles, 2012): (1) uncertainty sampling, which measures the uncertainty of the model's prediction on data points; (2) query by disagreement, which trains different models and checks their differences; (3) error and variance reduction, which directly looks into the generalization error of the learner. Besides, there are many other variants, such as density or diversity methods (Settles and Craven, 2008; Yang et al., 2015), which consider the repressiveness (reflection on input distribution) of instances in uncertainty sampling, clustering-based approaches (Dasgupta and Hsu, 2008; Nguyen and Smeulders, 2004; Saito et al., 2015) which cluster unlabeled data and query the most representative instances of those clusters, and min-max framework (Hoi et al., 2009; Huang et al., 2010) which minimizes the maximum possible classification loss. More versatile methods include combining multiple criteria (Du et al., 2015; Wang et al., 2016; Yang and Loog, 2018), choosing strategies automatically (Baram et al., 2004; Ebert et al., 2012), and training models to control active learning (Bachman et al., 2018; Konyushkova et al., 2017; Pang et al., 2018).

In addition to asking the oracle to label instances, queries may seek for more advanced domain knowledge. A simple idea is to solicit information about features. For instance, besides instance-label queries, a text classifier (Raghavan et al., 2006) may query the relevance between features (words) and classes, e.g. “is ‘puck’ discriminate to determine whether documents are about basketball or hockey?” Then, the vectors of instances are scaled to reflect the relative importance of features. Another way is to set constraints based on high-level features (Druck et al., 2009; Small et al., 2011). The oracle may be queried on possibilities, e.g. “what is the percentage of hockey documents when the word ‘puck’ appears?” The learning algorithm then tries to adjust the model to match the label distributions over the unlabeled pool. Other methods to incorporate features include adjusting priors in naive Bayes models (Settles, 2011), mixing models induced from rules and data (Attenberg et al., 2010), and label propagation in graph-based learning algorithms (Sindhwani et al., 2009). Humans sometimes do a poor job in answering such questions, but it is found that specifying many imprecise features may result in better models than fewer more precise features (Mann and McCallum, 2010).

Although most of active learning work is on classification, the principals also apply to regression (Burbidge et al., 2007; Willett et al., 2006). Recent work focuses on leveraging other concepts for larger data sets (e.g., image data), such as deep learning (Gal et al., 2017), reinforcement learning (Liu et al., 2019), and adversary networks (Sinha et al., 2019).

#### Interactive Visual Analytics

Visual analytics (VA) is a field where information visualization helps people understand the data and concepts in order to make better decisions. One core in VA is dimension reduction which can be well addressed through machine learning (Sacha et al., 2016). Applying the interactive VA, which allows users to give feedback in the modeling-visualizing loop, will make machine learning tools more approachable in model understanding, steering, and debugging (Choo and Liu, 2018).

In the combination of machine learning and VA, human knowledge plays an indispensable role as interactions and feedback to the system (Choo et al., 2010). Interactions and feedback can happen either in the visualization part or the machine learning part. In the former part, visualization systems satisfy users' requirements through interacting with them and assisting users in having a better understanding of the data analyzed by some machine learning methods. For instance, principle component analysis (PCA) is a powerful machine learning method which transforms the data from the input space to the eigenspace and reduces the dimension of the data by choosing the most representative components. However, for many users, PCA works as a “black box” and it is difficult to decipher the relationships in the eigenspace. Interactive PCA (Jeong et al., 2009) provides an opportunity for the users to give feedback on system visualization, and these interactions are reflected immediately in other views so that the user can identify the dimension in both the eigenspace and the original input space. In the machine learning part, interactions and feedback from humans help machine learning methods to generate more satisfying and explainable results and make the results easier to visualize as well. A good example of utilizing human interactions in machine learning methods is the application in classification (Fails and Olsen, 2003; Ware et al., 2001). These interactive classifiers allow users to view, classify, and correct the classifications during training. Readers can refer to a recent comment (Holzinger et al., 2018) on explainable AI.

The mutual interaction between human knowledge and the visualization or the model is an iterative process. A better visualization leads the users to learn more practical information. After the users gain some knowledge regarding the model and data, they can utilize the knowledge to further improve the model and even the learning algorithm (Hohman et al., 2018). This iterative process has a steering effect on the model, which can be viewed as the parameter evolution in dynamical systems shown in Equation (4) (Dıaz et al., 2016; Endert et al., 2017):

(Equation 4) $$\dot{y}=f\left(y,u\right),\;v=g\left(y\right),$$

where y is the model under the machine learning algorithm, v is the visualization of that model, and u={x,w} is the input including the new input data x and users' feedback w. The feedback w is based on users' knowledge as well as the visualization v. Training of the model is complete when the dynamical system settles down at a certain equilibrium, $y^{*}$.

Some progress has been made in this interdisciplinary area to help machine learning become more accessible. Semantic interaction (Endert et al., 2012) is an approach that enables co-reasoning between the human and the analytic models used for visualization without directly controlling the models. Users can manipulate the data during visualization, and the model is steered by their actions. In this case, interactions happen with the help of visualization and affect both model and visual results. Interactive visual tools can also be built for understanding and debugging current machine learning models (Bau et al., 2019; Karpathy et al., 2015; Zeiler and Fergus, 2014). The language model visual inspector system (Rong and Adar, 2016) is able to explore the word embedding models. It allows the users to track how the hidden layers are changing and inspect the pairs of words. The reinforcement learning VA systems, DQNViz (Wang et al., 2018) and ReLVis (Saldanha et al., 2019), allow users to gain insight about the behavior pattern between training iterations in discrete and continuous action space, respectively. As the users explore the machine learning algorithms better, they can compare different methods and adjust the model faster.

In the meanwhile, increasing the understandability of machine learning makes those algorithms more trustworthy and actionable and extends the application to more areas. An example is visualizing CNNs for autonomous driving where visualization serves as a debugging tool for real-time CNN-based systems. This is done by visualizing the regions of the input image which have the highest influence on the output (Bojarski et al., 2016). A recent paper (Endert et al., 2017) gives a summary of literature on how interactive VA is involved with the machine learning domains of dimension reduction, clustering, classification, and regression. It also shows some application domains in the field of integrating machine learning with VA, text analytics, and biological data analytics.

### Parameter Initialization

In essence, all the machining learning problems are optimization problems where we minimize the error/loss or maximize the benefit/probability from an initial start point. A bad initialization may lead to a slow converging path or even a sub-optimal result. In the following sections, we will introduce some works which apply human knowledge to initialization. We introduce how an agent learns from expert behaviors in Pre-training in Reinforcement Learning and how to use trained models by Transfer Learning. Although transfer learning is not limited to initialization, we categorize it here considering that fine-tuning pre-trained models is a widely used technique of transfer learning.

#### Pre-training in Reinforcement Learning

Many reinforcement problems must be solved in a large state/action space, especially for the continuous state action problems (Lazaric et al., 2008). Learning in a high-dimensional space requires huge amounts of data and learning time. Also, in many optimization methods, such as gradient-descent method and Newton's method, the initialization plays a critical role and may determine whether we are able to find the optimal value function/policy. Thus, equipping a pre-trained function as initialization in reinforcement learning becomes popular these days. Supervised learning is often used at the beginning stage of reinforcement learning, and the domain knowledge from humans is embedded in this way of initialization.

Pre-training can be applied to policy learning, value function learning, environment model learning, or a combination of them. In policy learning, humans can act as a trainer to teach agents the target parameterized policy through demonstration. A study provides a comprehensive survey of many different approaches to learning from demonstrations (Argall et al., 2009), and these approaches allow the agent to have a good initial policy before fine-tuning with interactive feedback. In the training of AlphaGO with DNNs and tree search (Silver et al., 2016), a supervised learning policy network is pre-trained directly from human expert moves. Then, a reinforce learning policy is trained to improve it by optimizing the final outcomes. In robot navigation, reinforce learning is capable of learning the fuzzy rules automatically but suffers from a heavy learning phase and insufficiently learned rules. Including supervised learning results as initialization in value function learning helps solve the issue and becomes one of the main approaches for this problem (Fathinezhad et al., 2016; Navarro-Guerrero et al., 2012; Ye et al., 2003). Learning a model of state dynamics can result in a pre-trained hidden layer structure that reduces the training time in reinforce learning problems (Anderson et al., 2015), and learning the deep Q networks from human demonstrators also helps to give a relatively good initial model and predict the dynamics (Gabriel et al., 2019). There are many other applications of smart initialization on policy gradient methods (Yun et al., 2017) and Q-learning methods (Burkov and Chaib-Draa, 2007; Song et al., 2012), which speed up the learning and level up the performance (Finn et al., 2016).

#### Transfer Learning

**Figure 5:**
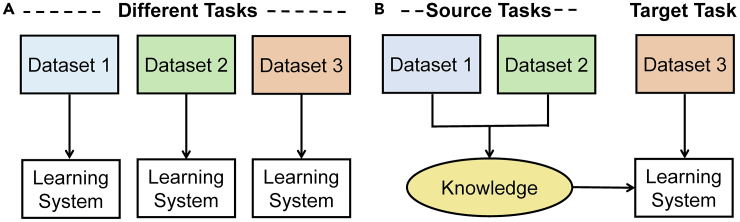
Illustration of Traditional Machine Learning and Transfer Learning (A) Tasks in traditional machine learning do not share knowledge. (B) Tasks in transfer learning share knowledge. Target task can reuse the knowledge of source tasks. Drawn based on the concept in (Pan and Yang, 2009).

Transfer learning is where knowledge is learned from a “source task” and applied to a “target task” (Pan and Yang, 2009). This method is inherently dependent on human knowledge to determine suitable source tasks to transfer. The transferred knowledge can be data, neural networks, weights, etc. The ideal situation to use transfer learning is when the source task has more data available than the target task. However, transfer learning can yield benefits even when the source task does not have as much data. As shown in Figure 5, traditionally in machine learning, the data is specific to the task being trained. By transferring knowledge from the already trained source task, less data specific to the target task are needed and the training time is reduced. The context provided from the source task increases the initial performance, rate of improvement, and final performance (Brownlee, 2017) while reducing the training time and the data needed. This efficiency has led to significant commercial use of transfer learning (Ruder, 2017b).

The role of human knowledge in transfer learning can be understood through the NLP problem of training a DNN for children's automatic speech recognition (ASR) (Shivakumar and Georgiou, 2020). There is a plethora of general speech data available, yet there is a lack of data specific to child speech recognition. It would be costly and time consuming to acquire data to train a child ASR algorithm from scratch. Instead, the data, neural networks, and weights from general ASR training can be transferred. Then, the weights can be fine-tuned for child ASR by using the limited amount of data specific to child ASR. The machine cannot know which source tasks would be beneficial to train the target task, so human knowledge is required to determine compatible and effective source tasks. Transfer learning is also used for image recognition where a human finds a source task to quickly train the target task before fine-tuning it with new visual data. This has been used in a wide variety of applications such as plant identification (Ghazi et al., 2017), structural damage detection (Gao and Mosalam, 2018; Gopalakrishnan et al., 2017), human behavior recognition (Kaya et al., 2017; Sargano et al., 2017), and even medical research (Burlina et al., 2017; Christodoulidis et al., 2016; Karri et al., 2017).

New methods of incorporating human knowledge with transfer learning are being researched to achieve higher efficiency and broader application. One of them is using simulation data. As we have mentioned in Section Simulation, machine learning can be trained with simulations before being fine-tuned with real-world data.

Another method that benefits from human knowledge is “heterogeneous transfer learning” (Day and Khoshgoftaar, 2017). Most commercial transfer learning methods are homogeneous, which means that the source and target tasks have the same feature space. For instance, in computer vision, the simulation looks different from the real world but the feature space is the same since they are both inputting pixels. In heterogeneous transfer learning, the feature spaces are different. For instance, the inputs are texts of two different languages, or one input is texts while the other is images. Heterogeneous transfer, by correlating different feature spaces, allows the source task to come from a wider variety of data. Extensive human knowledge on the subject is required to correlate the feature spaces effectively. The complexity of this correlation makes it difficult to scale heterogeneous transfer learning for broad use because each application of heterogeneous transfer learning requires a different subject of human knowledge. Other heterogeneous transfer learning techniques are being attempted to solve this problem, such as deep semantic mapping (Zhao et al., 2019) and hybrid heterogeneous transfer learning (Zhou et al., 2014, 2019).

Lastly, the importance of human knowledge for transfer learning is apparent by the poor performance that can occur without it. Sometimes the performance may actually be worse than if the target task was trained alone. This is known as negative transfer and occurs when the source tasks are poorly suited for the target tasks (Wang et al., 2019). It appears in human learning as well, e.g., learning to throw a baseball may be harder after learning to throw a football due to muscle memory making it difficult to adapt to a new throwing motion. Currently, preventing negative transfer requires effective human intuition or experience. Research is conducted to develop methods that will quantitatively eliminate negative performance, including using a discriminator gate to assign different weights to each source task (Wang et al., 2019) and using an iterative method that detects the source of the negative transfer to reduce class noise (Gui et al., 2018).

## Conclusions

In this paper, we give a comprehensive review on integrating human knowledge into machine learning. Knowledge is categorized into general knowledge and domain knowledge, and its representations are introduced together with the works that leverage them. We focus on some new and popular topics and group the methods by their major contribution to the machine learning pipeline. In conclusion, based on existing methods, we propose the following suggestions on improving the machine learning performance with knowledge:

1. Devise the inputs and outputs of models to make better use of resources. Aggregate tasks to learn together by Multitask Learning if they share data or information; an auxiliary task can be attempted even if only a single task is important. Use Features that could best represent the essence of tasks; the features can be manually engineered, selected by statistic metrics, or automatically learned by machine learning models.
2. Examine model assumptions to capture major factors. Set Variable Relation such as independence based on prior knowledge. The Distribution of unknown variables in models can be obtained by empirical data, expert intuition or Gaussian. Try to match the distribution of training data with test scenarios.
3. If using neural networks, tailor the architecture to be suitable for the tasks. If possible, incorporate some known properties, such as Symmetry of Convolutional Neural Networks. Logic, equations, and temporal nature can be, respectively, reflected in the structure of networks by, for instance, combining with symbolic AI, designing special layers/architectures, and using RNNs.
4. Augment data to incorporate invariant properties or knowledge-based models. Augmentation can be done by transforming, manipulating, or synthesizing the original data. Image and Audio data have been discussed in details, and their augmentation share similar principles. Simulations built upon knowledge-based models can be used to generate data.
5. Design algorithms to include humans in the loop. The interaction between machine and environment can be modeled and optimized in Reinforcement Learning . Humans can be asked to label data or provide distribution (Active Learning). Interactive Visual Analytics can be used to help humans understand machine learning results and then adjust models during or after training.
6. Find better initialization to reflect known results. This could be achieved by learning from expert behaviors before allowing machine to automatically learn from the environment (Pre-training in Reinforcement Learning). Transfer Learning can be used to distill knowledge from relevant tasks.

For future works, we highlight the following directions:

1. Models are dedicated and specific to tasks rather than universal. We witnessed the emergence of CNNs for images and RNNs for natural language. They are intuitively and empirically better than fully connected networks. General knowledge inspires us to leverage math and brains to propose more efficient mechanisms, such as attention (Vaswani et al., 2017). Domain knowledge captures the nature of the tasks, and more customized components can be incorporated.
2. More nodes are added to end-to-end learning for human interaction, feedback, and intervention. Despite convenient data preparation, the black box approach of end-to-end learning makes it difficult to explain and control. We can regulate the intermediate results or network layers (Zhang et al., 2018) to produce models more understandable and controllable by humans.
3. Existing results are reused for new targets. Humans can use the skills and insights across multiple tasks and even disciplines; some abilities are innate. Similarly, machines do not need to be trained from scratch. Given a new task, we can transfer or distill the knowledge from previous tasks or models.
4. Higher level features such as conceptual understanding and math theorems are incorporated. Currently, the knowledge integrated in machine learning is relatively concrete and mostly at the instance level, e.g. expressing each theorem as a constraint. Despite the efforts and achievements to make knowledge generic and broad, we have not seen a successful model to grasp abstract concepts or systematic theories. We believe integrating higher level features is an essential path toward strong artificial intelligence and would change the paradigm to integrate knowledge.

Designing and implementing machine learning algorithms is an iterative process. This requires humans to analyze the models and knowledge integration to take advantage of human understanding of the real world. This review may help current and prospective users of machine learning to understand these fields and inspire them to build more efficient models.
